# Developing a remote gamma-ray spectra collection system (RGSCS) by coupling a high purity Germanium (HPGe) detector with a cosmicguard background reduction device

**DOI:** 10.1016/j.ohx.2024.e00513

**Published:** 2024-02-02

**Authors:** Zaijing Sun, Krishnakumar Divakar Nangeelil, Haven Searcy

**Affiliations:** Department of Health Physics and Diagnostic Sciences, University of Nevada, Las Vegas, NV 89154, USA

**Keywords:** Automatic sampler, Gamma-ray detector, Remote Gamma Spectrum Collection System, CosmicGuard System

## Abstract

Despite being widely used for high-resolution spectral analysis and quantifying low activity in natural samples, the operations and data analysis of High Purity Germanium (HPGe) gamma-ray detectors are seldom fully automated due to the excessive costs associated with commercially available automatic sample changing systems. This paper introduces the design and implementation of a cost-effective, customized remote gamma-ray spectra collection system centered around the HPGe detector coupled to a cosmic-ray veto background reduction device. The HPGe detector system, equipped with a Lynx DSA, is seamlessly integrated with an economically viable automatic sample changer. This sample vial changer is controlled by a high-torque NEMA 34 stepper servo motor from Vention. Web control of the rotary actuator is facilitated through a CAD-based programming tool. The remote-controlled sample pick-and-place procedure is executed using a robotic arm (Trossen Robotics, Viper X 250). The DYNAMIXEL servomotors of the robotic arm are programmed using Python software supported by the Robotic Operating System. Beyond its technical construction, this system is uniquely fashioned for academic research, providing invaluable hands-on experience in gamma spectrometry to both junior researchers and students.

## Graphical abstract

**Figure:**
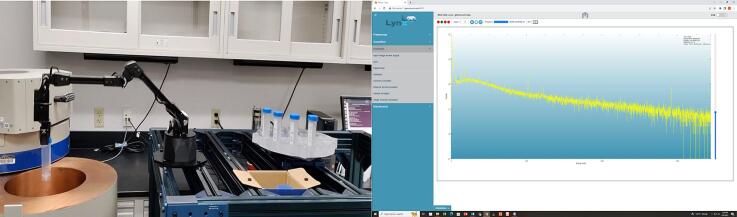

Specifications table.

**Table:** 

Hardware name	Remote Gamma-ray Spectra Collection System
Subject area	● Educational and research tools and open-source alternatives to existing infrastructure
Hardware type	● Other [Mechanical and Computer engineering]
Closest commercial analog	Automatic sample changer gamma spectrometry system
Open-source license	GNU General Public License (GPL)
Cost of hardware	$86,251.00, including the HPGe detector system
Source file repository	Figures are attached to the manuscript.

## Hardware in context

Given their high sensitivity, resolution, and reliability, High Purity Germanium (HPGe) detectors are widely used in radiation detection research and a variety of non-destructive analytical applications. Various investigations in the fields of environment, geology, and radiation sciences have shown that there is a growing need for HPGe gamma spectrometry in radiation-related research [1], [2]. Significant advances in HPGe detector fabrication technology over the past decade have resulted in improved crystal characteristics and detector performance. Features such as coaxial crystals and high relative efficiency have significantly enhanced sensitivity, efficiency, and energy resolution. Particularly in the fields of environmental and geological sciences, the widespread adoption of HPGe detectors has contributed to significant advancements [3], [4], [5].

The disruptions brought about by the COVID-19 pandemic have posed considerable challenges for students, researchers, and instructors who rely on laboratory-based investigations, especially in academic research environments [6], [7]. Measures like social distancing and restricted laboratory access have been crucial in curbing the spread of the virus. However, these measures have inevitably led to the suspension of numerous experiments, particularly those requiring close physical interaction or shared laboratory spaces. This interruption has not only hindered ongoing research but has also posed obstacles to the learning and teaching processes within educational institutions. To address these formidable challenges, a potential solution involves the development and deployment of a customized gamma spectroscopy system with an automatic sample changer tailored specifically for the academic research environment. Such a system could ensure the timely completion of research and maximize the system's capacity utilization. Commercially available auto sample changing systems from various manufacturers can aid in reducing detector idle time between frequent short acquisition measurements or enable unattended measurements for longer acquisitions, especially in counting laboratories with high volume sample processing requirements, thus minimizing the need for personal interventions [8], [9]. Many low-cost, state-of-the-art, and highly automated configurations of the HPGe gamma spectrometry system, tailored to diverse requirements, have been documented in the literature [10], [11]. However, this remote gamma-ray spectra collection system is specifically designed for customization within an academic environment for research and teaching.

The automation of operations of the HPGe detector systems has been mainly impeded by the high cost of commercial autosamplers and the need for substantial shielding to ensure proper system functionality. To overcome these challenges and effectively implement automation, the most viable solution appears to be the development of a Remote Gamma Spectra Collection System (RGSCS) that leverages the capabilities of the Internet for gamma spectral analysis. This system capitalizes on the networking potential of nuclear instrumentation. Its primary objective is to acquire and analyze gamma spectra over the Internet, which would greatly facilitate various research studies as well as student laboratory activities for nuclear sciences.

## Hardware description

**Fig. 1:**
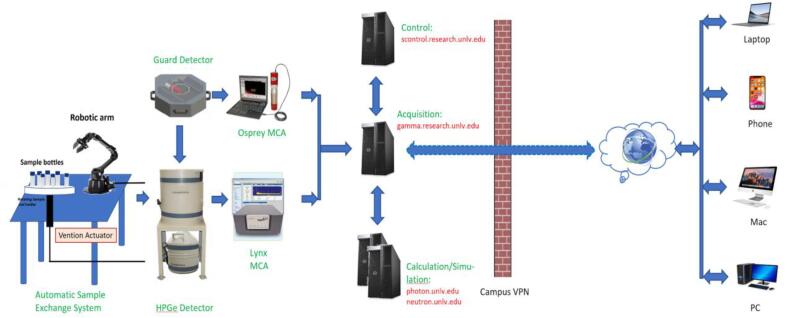
Design Overview of the Remote Gamma-ray Spectra Collection System.

The design overview of the RGSCS is depicted in Fig. 1. The RGSCS comprises an indigenous automatic sample changer system that is linked to the CosmicGuard HPGe gamma spectrometer, in addition to its associated structural and electronic components. These systems operate using separate network addresses in the secured university network. The integration of the automatic sample changer with the gamma spectrometry system and its network connectivity offers a high level of automation and convenience in the spectra acquisition process.

### The CosmicGuard gamma spectrometry system

The CosmicGuard gamma spectrometry system comprises a cryogenically cooled P-type closed-end coaxial HPGe detector (Model GX 3519, Mirion Technologies) with a relative efficiency of 35 % and a resolution of 1.9 keV for ^60^Co gamma energy at 1332 keV [12]. The Lynx digital signal analyzer (DSA) module from Mirion Technologies serves as the spectroscopy workstation for the spectral acquisition from the HPGe detector. This all-inclusive module encompasses crucial functionalities such as signal conditioning, rapid analog-to-digital conversion (ADC), programmable digital filters, automatic pole-zero and baseline restoration, High Voltage (HV) module, and a 32 K spectral memory [13], [14]. The Lynx module diligently processes signals received from the HPGe detector using an ultra-fast Analog-to-Digital converter, and subsequently, the obtained digital data is stored in the integrated multi-channel analyzer (MCA). The Lynx DSA is capable of doing spectral display and manipulation, spectrum analysis, and reporting on the host workstation using the Genie 2000 spectroscopy software from Mirion [15]. Additionally, the Lynx system supports remote access via a secured network for remote spectral analysis.

**Fig. 2:**
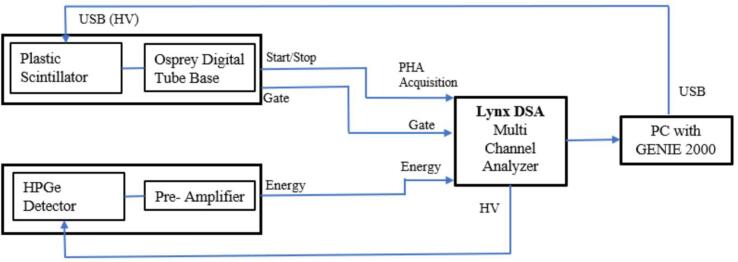
Schematic diagram of the HPGe cosmic veto system.

Cosmic radiation, constituting high-energy charged particles such as muons, poses a unique challenge as passive lead shielding proves inadequate in its attenuation. This radiation, predominantly oriented vertically, is mitigated through the implementation of a guard detector placed atop the lead shield. This specialized plastic scintillator, measuring 49.5 x 49.5 x 5 cm^3^, is employed in conjunction with the HPGe and Lynx DSA to suppress the cosmic background. Fig. 2 illustrates the schematic of the Cosmic veto detector system, elucidating the pertinent connections to the Lynx DSA module. Cosmic rays generally interact with the plastic veto detector positioned outside the top of the lead shield and the latter with the HPGe detector located within the lead shield, due to their high-energy characteristics. The plastic scintillator detector generates a gate signal to Lynx DSA whenever it detects a pulse. The precise alignment of the width and relative positions of the external pulse originating from the plastic scintillator with the HPGe detector store pulse is achieved through an integrated digital oscilloscope within the Lynx DCA firmware or Genie 2000. Consequently, the application of anticoincidence logic facilitates the acquisition of a gated spectrum, effectively excluding pulses arising from the simultaneous interaction with both detectors. This interaction leads to selective data processing, disregarding counts from the HPGe detector when both detectors sense radiation simultaneously. This approach significantly diminishes cosmic background influence in the high cosmic background laboratories [16].

### Automatic sample changer

**Fig. 3:**
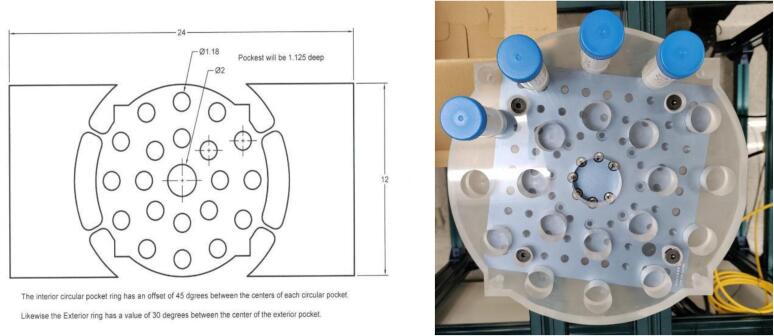
Sample vial holder.

A multiple-sample holder assembly is devised to accommodate 20 sample vials arranged in two circles, utilizing clear Acrylic material with a thickness of 1.25 in.. This assembly is affixed to a rotary actuator, which in turn is connected to a stepper servo motor. The design illustration of the sample holder assembly is presented in Fig. 3.

**Fig. 4:**
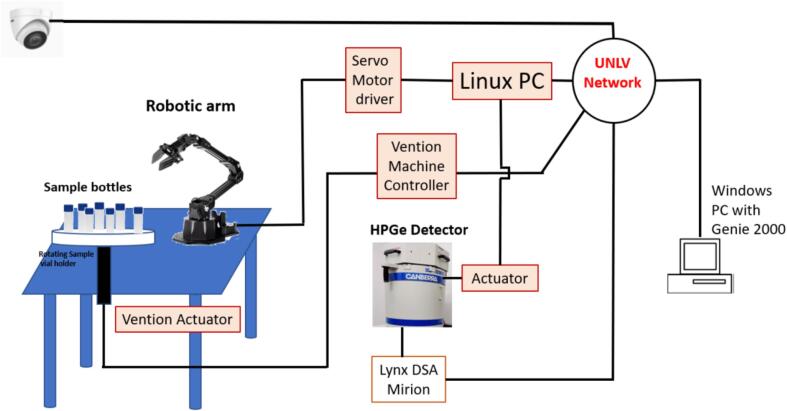
Schematic diagram of automatic sample changer for HPGe spectrometry system.

The rotary actuator with sensor (Part No. MO*-*RM*-*002*-*0001_3) from Vention offers unlimited 360-degree motion and drives the acrylic sample holder assembly fixed on the top of it. The rotary motion of the sample holder is precisely controlled by a 100 mm NEMA 34 stepper servo motor (97MM, 72Nm) and a machine motion controller (Vention Inc.) [17]. The NEMA 34 stepper servo motor (Part No. MO-SM-012–0000) used in this system is a high-torque motor that can deliver precise and smooth motion. The assembly encompassing the actuator, stepper motor, and Machine motion controller (Part No. CE-CL-010–0001) are affixed to a table structure made of Aluminum extrusions of varying lengths (for instance, Part No. ST-EXT-002–0765) and joint Aluminum assembly plates to reduce the cost. The Machine controller is configured with a static IP address for network connectivity and includes inputs for operating the servo stepper motor connected to the system, along with the safety stop and reset module (Part No. CE-SA-007–0000). The positioning of the sample vial holder is programmed utilizing the machine builder software (Vention Inc), which operates on a CAD-based platform designed to ensure seamless movement of the rotary actuator. Access to the machine controller is established over the Internet subsequent to configuration within the university's network. The network connectivity to the secured UNLV network enables users to input parameters such as sample counting time, delay, and the desired number of samples to be counted sequentially. Following the designated time interval, the sample vial assembly rotates to the subsequent position, facilitating the robot arm in grasping the subsequent sample. The schematic depiction of the Automatic Sample Changer is shown in Fig. 4.

### Robotic arm

**Fig. 5:**
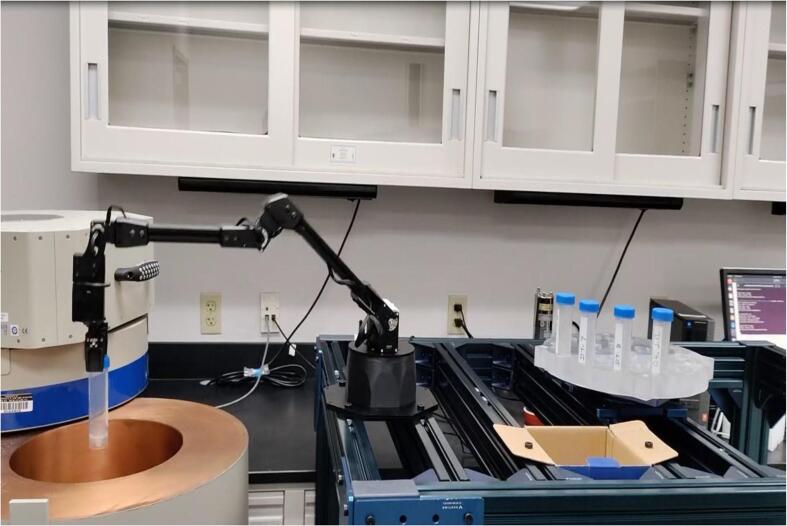
Remote Gamma-ray Spectra Collection system (RGSCS) in operation.

The robot arm is an essential component of the mechanical system that performs the crucial task of picking up the sample vial from the sample holder and placing it on the detector endcap to acquire the gamma-ray spectrum. The robot arm used for the system is Viper X 250 from Trossen Robotics [18]. It has 5 degrees of freedom and operates on 7 servo motors, providing precise and smooth movements. The robot arm is compatible with the open-source Robotic Operating System (ROS), which is a software library used for low-level device control, implementation of commonly used functions, and message passing between processes. To pick up the sample vial, the robot arm is programmed to move to the position of the vial, align its gripper, and close the gripper to grip the vial. The robot arm then moves and places the sample vial on the detector endcap to start the acquisition of the gamma-ray spectrum. After the preset time interval, the robot arm removes the sample vial from the detector endcap and places it in the dump basket or back to the sample holder, depending on the initial programming. The robot arm utilizes smart servos with multiple registers to set velocity and acceleration limits, PID gains, and more, which help fine-tune smooth joint motions. The DYNAMIXEL servo motors provide five degrees of freedom for the robotic arm, and low-level libraries are provided to abstract away the serial communication layer. This allows the user to program the trajectory using higher-level open-source programming languages such as Python.[19]. Fig. 5 shows the robotic arm is in operation in the laboratory.

**Fig. 6:**
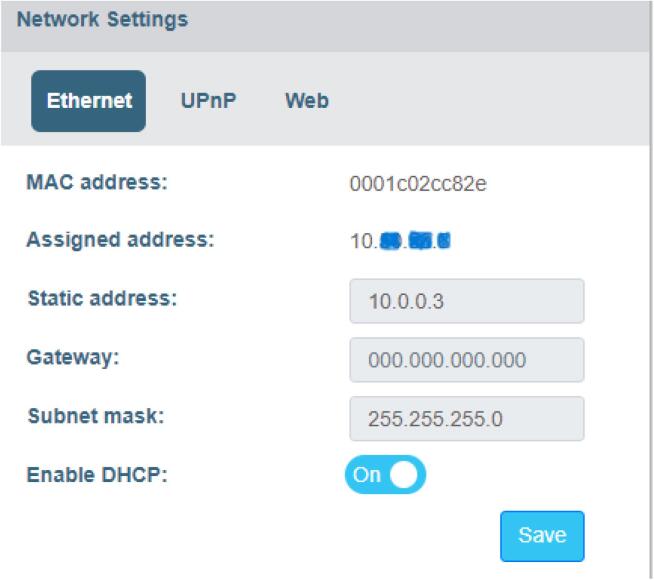
Lynx DSA network settings.

**Fig. 7:**
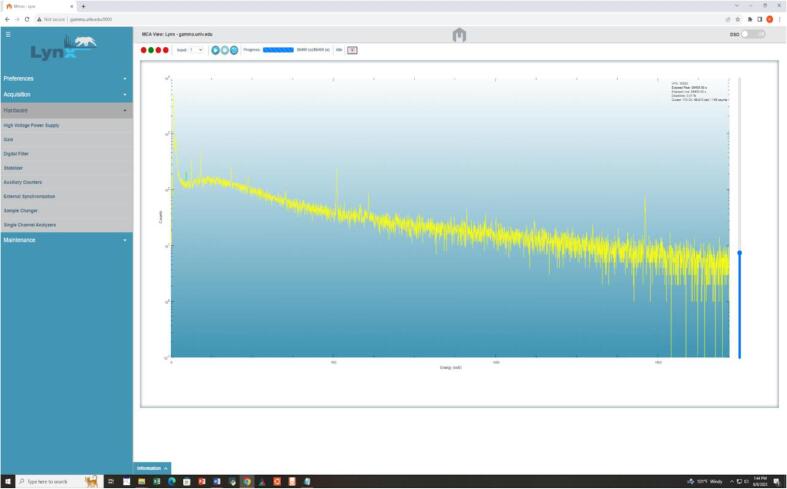
A typical background gamma spectrum acquired through RGSCS through web interference.

**Fig. 8:**
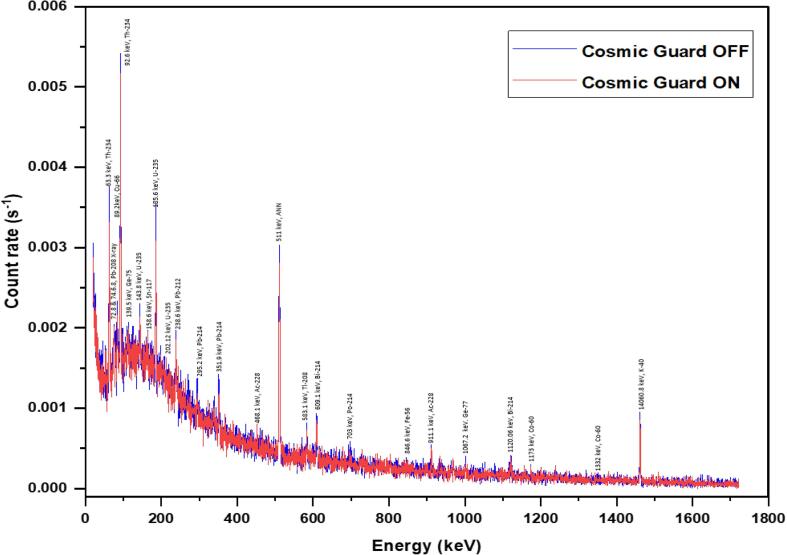
Remote gamma-ray spectra obtained for empty vial with CosmicGuard ON and OFF.

In summary, the RGSCS provides:

• Cost-effective alternative to commercially available automatic sample changing systems.
• Customized system to cater to academic environments by enabling online gamma spectra collection for both students and researchers, facilitating timely experiment completion and report writing.
• Potential applicability to any other gamma spectra collection laboratories utilizing NaI(Tl)/HPGe detectors.
• Safe handling of radioactive samples, minimizing exposure risks to users.

### Design files

All the design and schematic photographs of the RGSCS are available as Figures and included in the manuscript.

### Design files summary

**Table:** 

**Design file name**	**File type**	**Open-source license**	**Location of the file**
Fig. 1	Jpg	General Public License	Available with the article
Fig. 2	Jpg	General Public License	Available with the article
Fig. 3	Jpg	General Public License	Available with the article
Fig. 4	Jpg	General Public License	Available with the article
Fig. 5	Jpg	General Public License	Available with the article
Fig. 6	Jpg	General Public License	Available with the article
Fig. 7	Jpg	General Public License	Available with the article
Fig. 8	Jpg	General Public License	Available with the article

The figure comprises an overview of the RGSCS, an indigenous automatic sample changer system, linked to the Cosmic Guard HPGe gamma spectrometer from Mirion Technologies, in addition to its associated structural and electronic components for the remote gamma spectral collection. These systems use two separate network addresses in the university network.

The figure depicts the self-explanatory schematic of the Cosmic veto detector system, elucidating the pertinent connections to the Lynx DSA module.

A sample vial holder was designed and attached to the rotary actuator from Vention Inc. The sample vial holder is capable of accommodating a maximum of 20 samples in a single load.

The comprehensive structure of the automatic sample changer is depicted in this figure, providing a clear representation of the system's components and functionalities. The automatic sample-changing process is facilitated by a programmable sample holder driven by a stepper motor. The transfer of sample vials between the sample holder and the detector employs a robotic arm, which is an essential component of the automatic sample changer.

The figure shows the operation of the Remote Gamma Spectra Collection system in the laboratory. The robot arm removes the sample vial from the sample holder and places it on the detector endcap for counting/ spectra acquisition.

This figure shows the screenshot of the network setting configuration parameters for the Lynx DSA system for the university network.

A typical background spectrum acquired through networked Lynx DSA, offers a user-friendly interface that seamlessly integrates data acquisition and spectra analysis. The proprietary Lynx's web interface shows a vast array of powerful capabilities, including spectra acquisition, commencement, termination, data clearing, preset timing configurations, and the ability to store acquired spectra on a remote computer.

Gamma-ray spectra of an empty vial were acquired remotely under both CosmicGuard detector ON and OFF scenarios. The spectra were subsequently downloaded to a remote computer in CSV file format and visualized using Origin software. An evident 5 % reduction in cosmic background was observed in the background counts when CosmicGuard was activated.

### Python code

Python code developed for the robot arm operation involves defining the positions of the robot arm in the three-dimensional space in terms of the degrees of freedom provided by the servo motors. These positions are then used to program the movement of the robot arm for pick and place operations of the sample vials. The use of the robot arm and the programming of its operation are crucial aspects of the RGSCS, ensuring the efficient and accurate counting of the sample vials. The Python-ROS API is used to program the robot arm for pick and place operations of the sample vials. The Python code for the operation of the robot arm is attached in the supplementary materials.

### Electronics

The electronic circuit of the robotic arm is responsible for the smooth trajectory of the X, Y, and Z motions of the servo motors. It provides the necessary communication interface between the robotic arm and the computer via a USB cable, ensuring efficient and precise operation. This interface is easily accessible from any remote computer in the secured UNLV network, enabling remote control of the robotic arm. The user-friendly interface offers a simple and efficient way to control the robotic arm and handle the samples with ease.

#### Bill of materials

The bill of materials is submitted alongside the manuscript as supplementary material.

## Bill of materials summary

**Table:** 

**Designator**	**Component**	**Number**	**Cost per unit -currency**	**Total cost -currency**	**Source of materials**	**Material type**
Rotary table structure for robot arm and sample vial changer	Rotary table for Lab RobotME-OT-201331 v24	1	8543	8543	Vention	MetalandElectronics
Robot Arm for sample exchange	ViperX-250	1	4295	4295	Trossen Robotics	Electronics
Heavy Duty Actuator for moving the lead shielding top	Model PA 17–40-2000	1	500	500	Progressive Automations	Electronics
IP camera	Model No.SV-SB4-N	1	159	159	CCTV Security Pros	Electronics
Gamma server	Dell computer	1	1443	1443	Dell	Electronics
HPGe detector	ModelGX 3519	1	35,614	35,614	Mirion	Detector and Electronics
Cosmic veto System	CosmicGuard	1	35,697	35,697	Mirion	Detector andElectronics

## Build instructions

1. The assembly of the table (depicted in the figure within the Bill of Materials) necessitates Aluminum extrusions and joint Aluminum plates. These components are methodically combined by sequentially integrating the extrusions, adhering to the specified design.
2. Employ T-nuts judiciously to effectively secure distinct extrusion components in place.
3. Utilize a screwdriver to affix the Aluminum extrusions in accordance with the illustrated figure and the provided general assembly instructions accessible at: https://vention.io/resources/guides/vention-hardware-architecture-38.
4. Secure the sample vial holder onto the rotary actuator utilizing screws.
5. Integrate the rotary actuator with a servo stepper motor, which is managed by the machine motion controller.
6. Configure the machine motion controller to connect to the network using a static IP address.
7. Attach the robotic arm to one end of the table structure, adjusting its length to ensure unobstructed access within the detector shield.
8. Establish a connection between the robotic arm and the PC via a USB cable, enabling remote execution of the Python program.
9. Establish a connection between the HPGe Lynx DSA and the UNLV network.

## Operation instructions

1. Load the samples into the rotating sample vial holder within the laboratory.
2. Access the web interface of the automatic sample changer using any browser. Enter the counting time for each sample and initiate the program.
3. In the Python code for the robot, input the number of samples and the corresponding counting time for each. Execute the program.
4. Establish a connection to the Lynx DSA of the HPGe detector via its domain name. Input the predetermined counting time for the samples and commence data acquisition.
5. Save the gamma spectrum onto the local PC. Analyze the spectrum using Genie 2000, a software from Mirion. Alternatively, save it in CSV format on a remote computer.
6. Safety hazards are managed through the utilization of an emergency e-switch located on the sample vial table. If any malfunction occurs, the safety stop button is triggered, halting system operation. A manual release option for the safety button is also integrated, linked to the machine controller of the Automatic sample changer.
7. A video illustrating the system's functionality within a laboratory setting has been uploaded.

## Validation and characterization

### Data acquisition through web GUI

The process of analyzing the acquired spectra within the Lynx DSA system involves the utilization of specialized spectroscopic software called Genie 2000 from Mirion. This software is executed on a connected workstation or laptop and directly interfaces with the system, offering a wide range of analysis options tailored to specific application requirements. The Lynx system supports Ethernet (TCP/IP) network connections, as well as USB and serial RS-232C control. The default factory settings for the Lynx system are as follows: IP address 10.0.0.3 with a subnet mask of 255.255.255.0 [20]. To set up the Lynx system for use in the UNLV network, the following steps were taken:

1. Initially, the Lynx box was connected directly to the PC, and an internet browser on the PC was used to access its interface.
2. After accessing the Lynx interface, login credentials specific to the Lynx system were used to gain access.
3. Since UNLV operates on a DHCP network, changes were made to the network settings within the Lynx module to obtain a network address. This involves configuring the Lynx system to dynamically acquire an IP address from the UNLV network.
4. The MAC address of the Lynx system was directly associated with an IP address within the UNLV domain, ensuring a unique and recognizable identity on the network.
5. After configuring the new network parameters for the Lynx box, the system was connected directly to the UNLV network, allowing it to seamlessly communicate and share data within the university's network infrastructure.

These steps ensured that the Lynx DSA system was integrated into the UNLV network, allowing for effective analysis and data sharing in a networked environment. The network settings parameters for Lyx DSA are shown in Fig. 6.

The networked Lynx DSA system offers a user-friendly interface that seamlessly integrates data acquisition and spectrum analysis. The system's web interface provides a wide range of powerful capabilities, including spectrum acquisition, start and stop commands, data clearing, preset timing configurations, system diagnostics, instrument setup, and the ability to store acquired spectra as CSV files on a remote computer. Additionally, the system allows for remote control of the HV (High Voltage) bias, ensuring convenient operation. The system provides seamless integration of data acquisition and spectrum analysis, eliminating the need for multiple tools or complex setup procedures. An illustrative background spectrum obtained remotely is showcased in Fig. 7, effectively highlighting the system's capabilities and the advantages it brings in terms of efficient data acquisition, analysis, and remote accessibility. The High Voltage (HV) is set at + 4000 V with a rise time of 5.6 μs and a flat top of 0.8 μs, while the live time for spectral acquisition was configured at 86,400 s (24 h). The observed dead time for the detector during acquisition stood around 0.01 %. Therefore, the Lynx DSA, due to its user-friendly web interface and its ability to seamlessly integrate various functionalities, along with remote data storage and diagnostics, emerges as a valuable tool for researchers and students across various fields where spectral data analysis plays a critical role.

### CosmicGuard background reduction

Remote-recorded spectra can be downloaded in the form of CSV files, which can then be conveniently visualized using tools like Excel or Origin. The CSV format is chosen for its simplicity and compatibility with various data analysis and visualization tools. A study was conducted to assess a system's performance under different conditions, such as the 'CosmicGuard' detector was activated and deactivated. To evaluate this performance, no radioactive samples were involved, which implies that the measurements were taken using an empty sample vial. Both spectra were acquired for a duration of 86,400 s (24 h), utilizing settings identical to those detailed in Section 7.1. Fig. 8 represents a graphical representation of the collected spectra when the CosmicGuard detector was turned on and when it was turned off. The visual comparison of spectra under different conditions is essential for understanding the system's behavior and evaluating the effectiveness of CosmicGuard.

The remote spectra, obtained as a CSV and/or Excel file and visualized using MS Excel/ Origin, exhibited a 5 % reduction in the background when the CosmicGuard detector was activated. This reduction might be attributed to the elimination of cosmic background by the CosmicGuard detector. The spectra of the empty vial did not reveal any significant peaks apart from the common background gamma-ray spectrum. Well-defined peaks from the natural decay series of ^232^Th and ^238^U, along with their daughter products, are evident in our gamma spectrometry system. Several factors may account for these observations. In our current system configuration, we employ the Mirion model 747E lead shield (4 in. thick), which could potentially carry impurities, introducing the likelihood of interference in our measurements. This introduces disparities in background spectra when compared to the ultra-low background lead shield, Mirion 777, which is 6 in. thick and has options to flush nitrogen to reduce the concentration of radon, thoron, and their daughters between the detector and lead shield. The distinctive lines in the background spectra collected using our system can be attributed, at least in part, to long counting duration. Furthermore, the laboratory environment itself may contain elevated concentrations of natural radioactive elements, particularly from the Uranium (U) and Thorium (Th) natural series and their respective daughter products. Poor ventilation in the laboratory may exacerbate these concentrations, possibly leading to a secular equilibrium with the daughter products. Collectively, these factors contribute to the distinct background lines observed in our spectral data.

The RGSCS is presently configured to measure isotopes with energies below 2 MeV. Consequently, isotopes exhibiting higher energies may necessitate a recalibration of the gamma spectrometry system. When estimating the activity of diverse isotopes post-sample irradiation in INAA analysis, corrections for decay, cascade summing, and peak interference become essential. Since activity calculations are isotope-related, these corrections are manually applied during activity calculations and are not integrated into the system software. Although the automatic sample changing system is equipped with a software alert system to notify users in the event of malfunctions, communication errors involving the robotic arm are not communicated to the user. The comprehensive performance monitoring of the automatic sampling system can be facilitated through the network camera associated with the system. The automated lead door opening and closing mechanism, designed using a Heavy-Duty Linear Actuator with a robust force of 2000 lbs and a 40-inch stroke length, coupled with a mechanical linkage mechanism, exhibited malfunctions in the control box due to the excessive current drawn by the actuator during operation. The challenges associated with the high current are currently being addressed. The low suppression ratio due to the cosmic guard system may be attributed to the low cosmic background in the laboratory. However, the primary use of the system in analyzing passive environmental sample analysis (vegetation, soil, water), and filter paper analysis from various Community Environment Monitoring Program stations, may necessitate a greater reduction in background to improve the Minimum Detectable Activity (MDA) of radioisotopes.

Another limitation of the system lies in its current incapability to count multiple samples for different durations. Overcoming this constraint requires essential software modifications to enable the system to count various samples for varying durations effectively.

## Ethics statements

All authors have read, understood, and have complied as applicable with the statement on “Ethical Responsibilities of Authors” as found in the Instructions for Authors. The authors declare no competing interests.

## CRediT authorship contribution statement

**Zaijing Sun:** Writing – review & editing, Writing – original draft, Validation, Supervision, Project administration, Methodology, Funding acquisition, Conceptualization. **Krishnakumar Divakar Nangeelil:** . **Haven Searcy:** Writing – original draft, Methodology, Investigation.

## Declaration of competing interest

The authors declare that they have no known competing financial interests or personal relationships that could have appeared to influence the work reported in this paper.
